# A Novel Class of Mitochondria-Targeted Soft Electrophiles Modifies Mitochondrial Proteins and Inhibits Mitochondrial Metabolism in Breast Cancer Cells through Redox Mechanisms

**DOI:** 10.1371/journal.pone.0120460

**Published:** 2015-03-18

**Authors:** Praveen K. Vayalil, Joo-Yeun Oh, Fen Zhou, Anne R. Diers, M. Ryan Smith, Hafez Golzarian, Patsy G. Oliver, Robin A. J. Smith, Michael P. Murphy, Sadanandan E. Velu, Aimee Landar

**Affiliations:** 1 Department of Pathology, University of Alabama at Birmingham, Birmingham, Alabama, United States of America; 2 Center for Free Radical Biology, University of Alabama at Birmingham, Birmingham, Alabama, United States of America; 3 Department of Radiation Oncology, University of Alabama at Birmingham, Birmingham, Alabama, United States of America; 4 Department of Chemistry, University of Alabama at Birmingham, Birmingham, Alabama, United States of America; 5 Department of Chemistry, University of Otago, Dunedin, New Zealand; 6 MRC Mitochondrial Biology Unit, Cambridge, United Kingdom; National Institute of Environmental Health Sciences, UNITED STATES

## Abstract

Despite advances in screening and treatment over the past several years, breast cancer remains a leading cause of cancer-related death among women in the United States. A major goal in breast cancer treatment is to develop safe and clinically useful therapeutic agents that will prevent the recurrence of breast cancers after front-line therapeutics have failed. Ideally, these agents would have relatively low toxicity against normal cells, and will specifically inhibit the growth and proliferation of cancer cells. Our group and others have previously demonstrated that breast cancer cells exhibit increased mitochondrial oxygen consumption compared with non-tumorigenic breast epithelial cells. This suggests that it may be possible to deliver redox active compounds to the mitochondria to selectively inhibit cancer cell metabolism. To demonstrate proof-of-principle, a series of mitochondria-targeted soft electrophiles (MTSEs) has been designed which selectively accumulate within the mitochondria of highly energetic breast cancer cells and modify mitochondrial proteins. A prototype MTSE, IBTP, significantly inhibits mitochondrial oxidative phosphorylation, resulting in decreased breast cancer cell proliferation, cell attachment, and migration *in vitro*. These results suggest MTSEs may represent a novel class of anti-cancer agents that prevent cancer cell growth by modification of specific mitochondrial proteins.

## Introduction

It has long been known that cancer cells are metabolically distinct from normal cells. For example, many cancer cell types exhibit higher levels of glycolysis, irrespective of the levels of available oxygen needed for oxidative phosphorylation, a phenomenon known as “aerobic glycolysis” or the Warburg effect [1]. This observation led to the concept that cancer cells have defective or disrupted mitochondrial metabolism. However, recent evidence demonstrates that many cancer cells do not have impaired mitochondrial function, but in fact have higher levels of ATP-dependent oxygen consumption compared with normal cells [2–4]. In addition, cancer cells which are resistant to standard therapeutic regimens have increased mitochondrial reserve capacity [5, 6]. Therefore, targeting cancer cell mitochondrial metabolism, particularly ATP-dependent oxygen consumption and reserve capacity, may potentially represent a novel approach to cancer treatment.

Mitochondria are critical for a number of cellular processes including energy generation, anaplerosis, regulation of apoptosis, redox cell signaling, and calcium homeostasis. A major strategy for targeting compounds to mitochondria involves the use of lipophilic cationic carrier moieties to deliver functional groups such as antioxidants and DNA alkylating agents. The triphenylphosphonium (TPP) moiety is the best characterized mitochondria-targeting group and has been used to deliver the antioxidant coenzyme Q, nitrogen mustards, doxorubicin, and other molecules to the mitochondrion [2, 7, 8]. This is accomplished via covalent linkage of molecular moieties to TPP to form bifunctional compounds. TPP-linked molecules have also been shown to disrupt mitochondrial function *in vitro* at high concentrations after short-term exposure [2, 7, 9, 10], though the precise mechanisms remain poorly defined.

In this study, we analyze the bioenergetic consequences of directing electrophilic TPP bifunctional compounds to the mitochondrion. These compounds, termed “mitochondria-targeted soft electrophiles,” (MTSEs), differ significantly in their reactivity from highly toxic electrophilic drugs and environmental toxicants, which are relatively “hard” electrophiles [11]. Hard electrophiles form adducts with “hard” nucleophiles such DNA bases and serine protein residues; whereas soft electrophiles form adducts with “soft” cellular nucleophiles, particularly cysteine thiols. While hard electrophiles have routinely been dismissed as therapeutics due to their systemic toxicity in drug studies, there is accumulating evidence that soft electrophiles are less toxic in *in vitro* and *in vivo* biological model systems [11, 12]. It is also important to consider that the soft electrophile class of compounds have a range of reactivity spanning several orders of magnitude [13]. The reactivity of a soft electrophile is also directly proportional to the toxic effects, with more reactive compounds exhibiting higher toxicity in cellular and animal models [14–16]. Therefore, it is likely that soft electrophiles of relatively low reactivity, including MTSEs, may be useful as therapeutic agents. In fact, other such soft electrophiles have known beneficial physiological effects and include dietary electrophiles found in broccoli (sulforaphane) and curry (curcumin) [17], as well as endogenously produced anti-inflammatory prostanoids such as 15-deoxy prostaglandin J_2_ [18, 19].

One of the most important considerations in developing novel drug leads is ensuring specific interaction of the compounds with desired target protein(s). In the case of electrophilic signaling molecules, the specificity of reaction is determined by the chemical properties of the molecules themselves, including hydrophobicity, reactivity, electrophile “softness,” and target “softness” [11]. In general, lower reactivity of the electrophile results in higher selectivity for specific targets. The most reactive soft nucleophiles within the cell are selenocysteine and deprotonated (or low pK_a_) cysteine residues [20, 21]. While cysteine is present in most proteins, it represents less than 2% of the total protein amino acid composition. In addition, not all cysteines are susceptible to oxidative modification, since relatively few cysteines exist primarily in the deprotonated, nucleophilic form [21, 22] which is reactive with electrophiles. It is for these reasons that specific protein thiols are poised to mediate diverse redox signaling responses to multiple stimuli [23]. Interestingly, accessible reactive protein thiols are present in the active sites of many mitochondrial proteins. Mitochondrial proteins are exposed to the most reducing environment within the cell and are susceptible to modification due to the relatively high inner mitochondrial matrix pH caused by the proton pumping of the electron transport chain [24]. Mitochondrial proteins which are redox-sensitive include mitochondrial dehydrogenases such as α-ketoglutarate dehydrogenase [25], isocitrate dehydrogenase [26], and mitochondrial aldehyde dehydrogenase [27], as well as the mitochondrial complexes I, II, and V [28, 29].

In order to determine the effects of mitochondrial protein modification on the metabolism of cancer cells, we synthesized a series of MTSEs that alkylate mitochondrial proteins and examined the differential effects of a prototype MTSE on oxidative phosphorylation and glycolysis in tumorigenic versus non-tumorigenic breast cells. In addition, we determined the resultant effects of MTSEs on breast cancer cell proliferation, migration and adhesion. This study demonstrates that MTSEs cause profound inhibition of mitochondrial metabolism, and inhibit breast cancer cell proliferation, attachment, and migration; while non-tumorigenic MCF10A cells remain relatively insensitive. Taken together, these results suggest that modification of mitochondrial thiols by MTSEs alters metabolic pathways in breast cancer cells, and this class of compounds may be useful to inhibit the progression of highly energetic cancer cells.

## Materials and Methods

All chemicals were of analytical grade and purchased from Sigma-Aldrich (St. Louis, MO) unless otherwise noted. Anti-TPP antiserum was prepared as previously described [30]. The MDA-MB-231 cell line was a generous gift from Dr. Danny Welch, and was originally obtained from ATCC. The MDA-MB-468 cell line was obtained from ATCC.

### Synthesis of mitochondria-targeted electrophiles

Mitochondria-targeted electrophiles were synthesized containing an iodo-leaving group with alkyl linkers of varying carbon lengths, including 3-, 4-, 6-, 8- and 10-carbons [iodopropyl triphenylphosphonium (IPTP), iodobutyl triphenylphosphonium (IBTP), iodohexyl triphenylphosphonium (IHTP), iodooctyl triphenylphosphonium (IOTP), iododecyl triphenylphosphonium (IDTP), respectively]. These analogs were synthesized as previously described [31]. Briefly, triphenylphosphine was heated at 100°C for 45 minutes with 1,3-diiodopropane, 1,4-diiodobutane, 1,6-diiodohexane, 1,8-diiodooctane or 1,10-diiododecane, respectively. After the reaction was complete, diethyl ether was added and the precipitated solid was filtered and dried. All compounds were characterized by ^1^H-NMR, ^13^C-NMR and MS in order to ensure purity of >99.9% before their use in biological evaluations.

### Cell Culture and Treatments

#### Breast cancer cell lines

MDA-MB-231 (MB231) and MDA-MB-468 (MB468) human breast adenocarcinoma cells were cultured in DMEM (Mediatech, Manassas, VA) supplemented with 10% fetal bovine serum (FBS; Atlanta Biologicals, Atlanta, GA). Cultures were maintained in 5% CO_2_ and humidified in a 37°C incubator. MCF10A, an immortalized human breast epithelial cell line, was obtained from American Type Culture Collection (ATCC, Manassas, VA). MCF10A cells were maintained in a mammary epithelial cell culture medium (MEGM) supplemented with MEGM SingleQuot bullet kit (Lonza,Walkersville, MD).

#### Cell Treatments

Cells were plated at a cell density of 2×10^5^ cells/well in 6-well plates and allowed to acclimate for 16h. For breast cancer cells, medium was changed to low FBS-containing medium (0.5%) for 16h prior to treatment. For MCF10A cells, supplemented MEGM medium was diluted 1:20 with non-supplemented MEGM for 16h prior to treatment. Cells were then treated with MTSE in 1mL of fresh medium for times indicated in the figure legend. At the end of treatment, cell lysates were prepared and protein adducts were visualized by Western blot analysis as described below under “Immunoblot Analysis.”

### Cell Fractionation

MB231 were plated in 6-well plates and allowed to grow until 90% confluent. Cells were divided into crude mitochondrial and cytoplasmic fractions by differential centrifugation. Briefly, cells were washed 2 times with phosphate-buffered saline (PBS), and each well from a 6-well plate was scraped into 167μL of MIB-1 (mitochondrial isolation buffer-1: HEPES, 50mM; sucrose 100mM; KCl 100mM; EGTA, 1mM; pH 7.2 with KOH). All 6 wells were combined to make one sample. Digitonin (5mg/mL in MIB-1) was added to a final concentration of 50μg/10^6^ cells and samples mixed by inversion for 1 min. Samples were centrifuged at 1000 x g for 10min at 4°C, and half of supernatant was transferred to a new tube. The pellet was homogenized with a micropestle with the remaining supernatant and centrifuged again at 1000 x g for 10min at 4°C. Supernatant was collected, pooled with the initial supernatant, and centrifuged at 14,000 x g for 10min at 4°C in order to pellet the mitochondrial fraction. Supernatant (containing cytoplasm components) was collected and used for analysis. Pellet containing mitoplast enriched fraction was washed once in MIB-2 (mitochondrial isolation buffer-2: prepared as above for MIB-1 with omission of EGTA) by resuspending pellet and repeating previous centrifugation step. Supernatant was discarded and mitoplast fraction used for analysis. Mitochondrial enrichment procedure was confirmed by Western blot analysis of the mitochondrial protein, the voltage-dependent anion channel. Protein adducts were visualized by Western blot analysis as described below.

### Immunoblot Analysis

Cells were washed with PBS and lysed in a buffer containing 10mM Tris-HCl, pH 7.4, and 1% Triton X-100 and protease inhibitor cocktail (Roche). Soluble proteins were resolved using SDS-PAGE and transferred to nitrocellulose membranes (Bio-Rad). Protein levels were quantified using the method of Bradford (Bio-Rad), and equivalent amounts of protein were loaded. Uniform protein loading was confirmed using Ponceau S staining of membranes and showed no significant differences in protein levels on blots among samples. Membranes were blocked in 5% milk (w/v) in Tris Buffered Saline (pH 7.4) containing 0.5% Tween 20 (TBS-T), and then incubated with anti-TPP (1:10,000) primary antiserum for 3h at room temperature. After washing with TBS-T, membranes were incubated with HRP-conjugated anti-rabbit IgG secondary antibody. Membranes were developed using SuperSignal West Dura chemiluminescence substrate (Pierce) and imaged using a CCD camera imaging system.

### Metabolic Assessment

To measure mitochondrial metabolism in intact MB231 cells, an XF24 Extracellular Flux Analyzer was used with a mitochondrial “stress test” [32]. The optimal seeding density of MB231 and MCF10A cells was determined to be 40,000 cells per well. The mitochondrial stress test uses sequential injections of oligomycin, FCCP and antimycin A to define a number of mitochondrial parameters such as basal OCR, ATP-linked OCR, proton leak, maximal respiratory capacity, reserve respiratory capacity and non-mitochondrial oxygen consumption, as described previously [33–35]. The concentrations of these compounds were optimized prior to the assay. In our calculations, the oligomycin-insensitive OCR was attributed to proton leak.

To determine the effects of MTSEs, cells were plated on XF24 plates and treated with the indicated concentrations of IBTP or BTPP for 4h in 0.5% FBS-containing medium. After treatment, the medium was removed and replaced with XF assay medium (DMEM, containing 5mM glucose, 0.5% FBS, 5mM HEPES without bicarbonate) and equilibrated 1h before OCR measurement. For 24h post-treatment measurements, cells were plated on 6-well plates and treated with the indicated concentrations of IBTP or BTPP for 4h. After the incubation, the cells were harvested immediately by trypsinization and replated into XF24 plates for an additional 20h in complete medium containing 10% FBS (total 24h). The medium was removed and replaced with assay medium and equilibrated 1h before OCR measurement. For 72h post-treatment measurements, cells were plated on 6-well plates and treated with the indicated concentrations of IBTP or BTPP for 4h. The medium was replaced with complete medium containing 10% FBS, and incubated for 48h. The cells were harvested after 48h, replated in XF24 plates and allowed adhere for an additional 20h in complete medium (total duration 72h). The medium was replaced with assay media and incubated 1h before measurement of OCR.

Glycolytic function was measured using a glycolytic “stress test.” For these experiments, cells were cultured and treated similarly to the mitochondrial stress test, except that prior to measurement medium was changed to DMEM without glucose, bicarbonate or HEPES, and containing 0.5% FBS. ECAR was monitored using the XF analyzer, and sequential injections of glucose (10mM), oligomycin (1μg/mL), and 2-deoxyglucose (2DG; 100mM) were used to determine glycolytic function parameters. Initially, ECAR was measured in the assay medium without glucose. The ECAR measured in the absence of glucose represents non-glycolytic acidification. Glucose was then added, which stimulates glycolysis and represents glycolytic rate or glycolytic flux in the cells. Oligomycin was injected to inhibit mitochondrial ATP production, and increase glycolytic rate in order to meet the energy demands of the cells. This increase represents the maximum glycolytic capacity of the cells. The difference in ECAR between maximum glycolytic capacity and glycolytic rate represents glycolytic reserve, which defines the maximum ability of the cells to induce glycolysis to meet the energy demands in the absence of mitochondrial ATP production. Both OCR and ECAR measurements were normalized to total protein per well.

### Cell Proliferation

Proliferation of MB231 cells was measured by using CyQuant cell proliferation assay kit from Molecular Probes (Eugene, OR). The basis for the CyQuant assay is the use of a proprietary green fluorescent dye (CyQuant GR dye) that exhibits strong fluorescence when bound to cellular nucleic acids. Cells (200,000/well) in 6-well plates were treated with different concentrations of IBTP for 24h in medium containing 0.5% FBS containing medium at 37°C in a CO_2_ incubator. Treatment was stopped by removing the media completely and replenishing with fresh media with 0.5% FBS without IBTP and incubated for another 48h. After incubation, media was completely removed and cells were frozen at −80°C then thawed and lysed according to the manufacturer’s protocol. The lysates (100μl) were diluted with an equal volume of CyQuant GR dye, and fluorescence was measured after 5 min using Victor X3 multi-well plate reader with excitation 485 nm and emission 530 nm.

### Cell Attachment and Migration

MB231 cells were plated at a cell density of 2×10^5^ cells/well in 6-well plates. Medium was changed to low FBS-containing medium (0.5%) for 16h. Cells were then treated with the indicated concentrations of IBTP or BTPP in 1mL of fresh medium containing 0.5% FBS for 4h.

To determine the effect of IBTP on cell migration, the cells were treated as described above. After the treatment, medium was changed to fresh 0.5% FBS-containing medium to remove compounds and scratches were made on the cell layer. The cells were allowed to migrate for 5h into cell free zone. Images of the scratches were captured at 0h and 5h. The length across the cell free zone before and after 5h of incubation was measured using ImageJ software (NIH, Bethesda, USA).

To determine the effect of IBTP on cell attachment, the cells were trypsinized after treatment, counted and the viability was assessed by trypan blue exclusion assay. The cells from each treatment group were then transferred to a 100-mm tissue culture dish and incubated for 24h. At the end of the incubation, the media was collected and total number of viable cells that failed to attach to the substratum was counted.

### Statistical analysis

Results are reported as means ± SEM, and n = 3 or more determinations as indicated in the Figure legends. Data were analyzed by one-way analysis of variance (ANOVA) followed by post-hoc analysis for significant results among the groups by pairwise t-tests using MegaStat Software. The minimum level of significance was set at p<0.05.

## Results

### Post-translational modification of proteins by MTSEs in breast cancer cells

**Fig 1:**
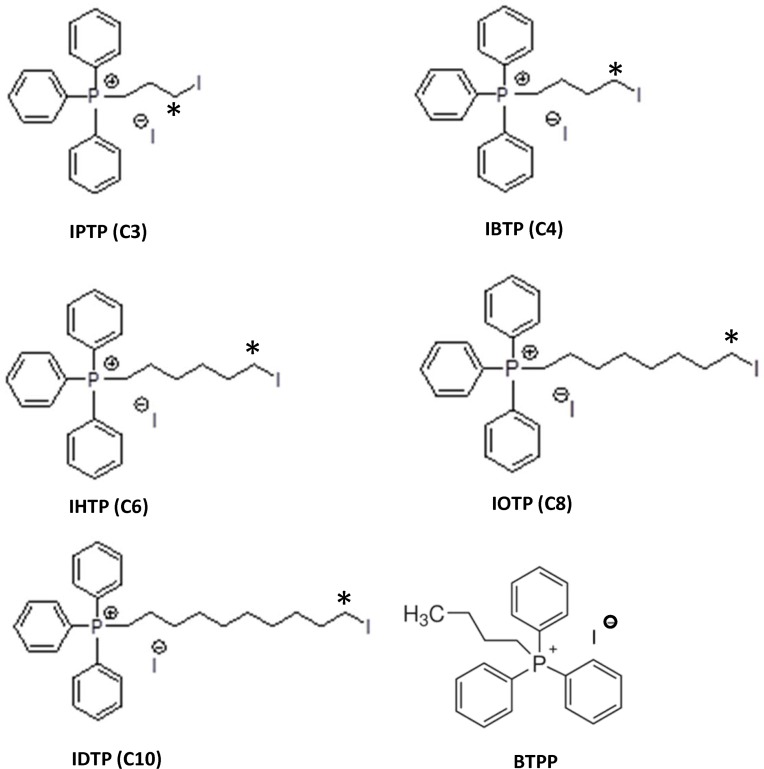
Structures of mitochondria-targeted electrophiles. The compounds were synthesized as described under “Materials and Methods.”

We hypothesized that MTSEs may represent a novel class of compounds which act by forming adducts with key metabolic proteins and ultimately inhibiting mitochondrial bioenergetics pathways. Therefore, analogs of IBTP were synthesized with varying chain lengths in order to optimize the hydrophobicity and accessibility of the electrophilic carbon adjacent to the iodo- leaving group (Fig. 1). Compounds include iodopropyl-, iodobutyl-, iodohexyl-, iodooctyl-, and iododecyl-triphenylphosphonium (TPP), and a non-electrophilic analog with a 4-carbon alkyl chain (BTPP) that can be used as a control for effects due to the TPP moiety independent of alkylation activity.

**Fig 2:**
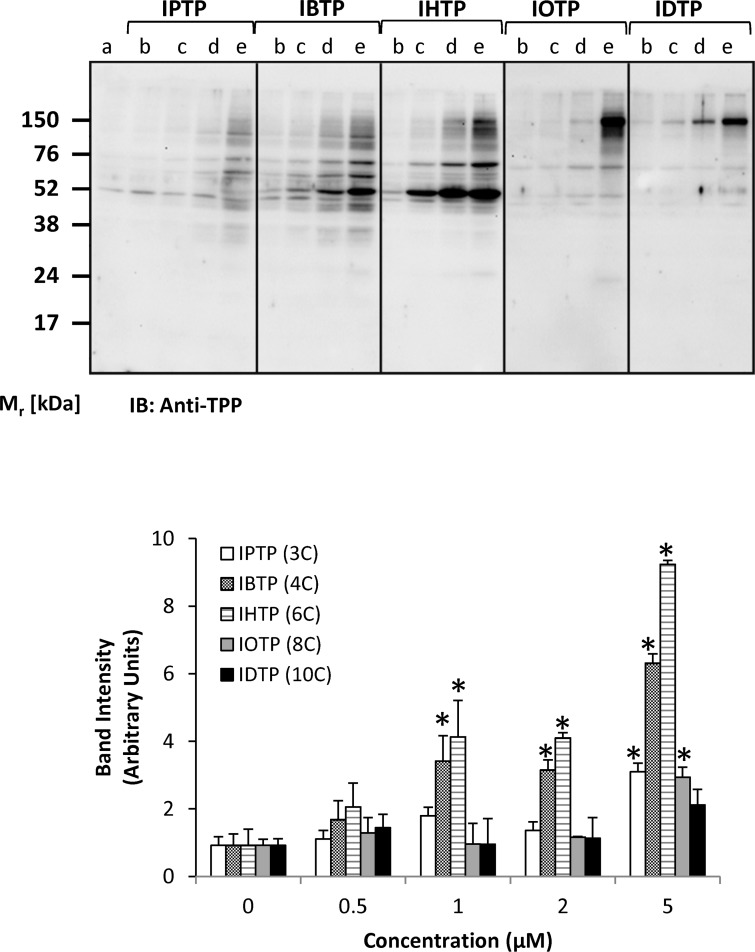
Dose-dependent modification of proteins by MTSEs of different chain length in MB231 cells. MB231 cells were treated with the indicated doses for 4h. At the end of treatment, cell lysates were prepared and protein adducts were visualized by Western blot analysis using an antibody directed against TPP. **a** = Vehicle; **b** = 0.5 μM; **c** = 1μM; **d** = 2μM; **e** = 5μM. IPTP = 3 carbons, IBTP = 4 carbons, IHTP = 6 carbons, IOTP = 8 carbons, IDTP = 10 carbons. The values are mean ± SE of 3–5 samples obtained from two separate experiments; *p<0.05 compared to no treatment.

**Fig 3:**
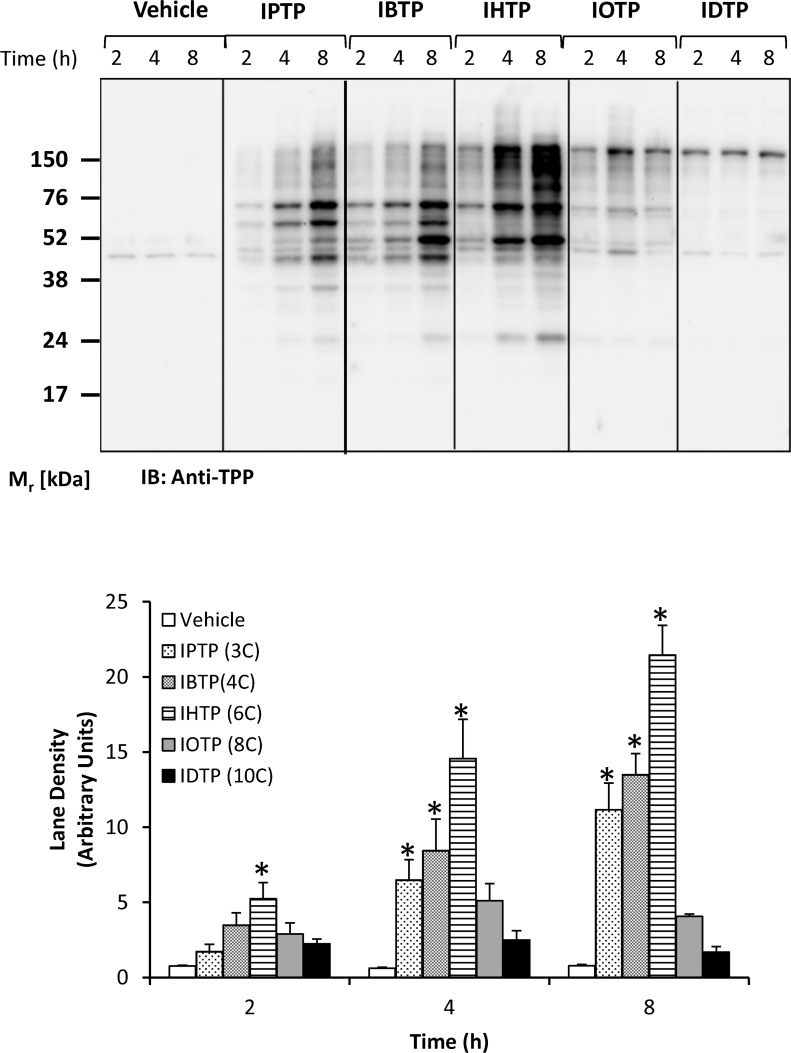
Time-dependent modification of proteins by MTSEs of different chain lengths in MB231 cells. MB231 cells were treated with 5μM of indicated MTSEs for the indicated times. At the end of treatment, cell lysates were prepared and protein adducts were visualized by Western blot analysis using an antibody directed against TPP (upper panel). Lane densities were quantified and plotted in the lower panel. Values are mean ± SE of 3–5 samples obtained from two separate experiments; *p<0.05 compared to no treatment.

In order to characterize protein adduct formation by MTSEs in breast cancer cells, MB231 cells were treated with MTSE compounds over a dose-range from 0–5μM, and adduct formation was determined by Western blot analysis with quantification (Fig. 2). Our results indicate that protein modification by MTSEs is dependent on the carbon chain length, with greater adduct formation observed with IBTP and IHTP compared with other analogs. Overall, there was a trend toward higher molecular weight adducts with increasing chain length. These results suggest that the predominant proteome modified by each compound may be distinct. For alkyl chains ranging from 3–6 carbons, TPP adducts also accumulate with time (Fig. 3). Interestingly, alkyl chains longer than 6 carbons did not cause time-dependent formation of adducts during the experimental period, suggesting that maximal accumulation and adduct formation occurred earlier than 2h. It is also possible that alkyl chains longer than 6 carbons may cause depolarization of the mitochondrial membrane potential, and subsequent failure to accumulate the more hydrophobic compounds. These results indicate that MTSEs with alkyl chain lengths ranging from 3 to 6 may be the better candidates for the development of cancer therapeutics. Thus, we chose to investigate IBTP containing a 4-carbon linker as a prototype MTSE in subsequent experiments.

### Accumulation of protein adducts in mitochondria in breast cancer cells

**Fig 4:**
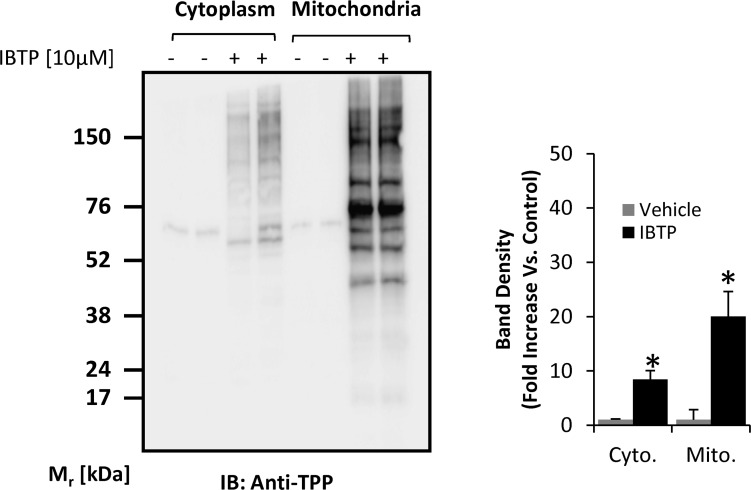
Mitochondrial protein modification by IBTP in breast cancer cells. MB231 cells were treated with 10μM IBTP or EtOH vehicle for 4h, and cell fractions were obtained as described in the Materials and Methods. At the end of treatment, cell lysates were prepared and protein adducts were visualized by Western blot analysis using an antibody directed against TPP. The values are mean ± SE of 3–5 samples obtained from two separate experiments; *p<0.05 compared to vehicle.

Because triphenylphosphonium compounds accumulate within the mitochondrion, IBTP treatment is expected to result in more protein adducts in the mitochondrial fraction than the cytoplasmic fraction, although this has not been demonstrated in cancer cells. MB231 cells were treated with vehicle, BTPP or IBTP over a dose range up to 15μM, and proteins containing triphenylphosphonium adducts were detected (S1 Fig.). No adducts were formed in the BTPP-treated cells, as expected. Adduct formation with proteins in cytosolic or mitochondrial compartments was determined by treatment of MB231 cells with 10μM IBTP or vehicle for 4h, followed by isolation of the mitochondrial and cytoplasmic fractions. The quality of mitochondria-enriched fractions was determined by Western blot analysis to confirm the presence of the voltage-dependent anion channel in the mitochondria fractions, and its absence in the cytoplasmic fraction (S2 Fig.). Differences in protein adduct formation in these fractions were determined by Western blot analysis. Fig. 4 shows an enrichment of protein adducts of approximately 2.3 fold in the mitochondrion when compared with the cytosolic fraction, clearly indicating that IBTP concentrates within the mitochondria and forms protein adducts.

### Effects of IBTP on cellular metabolism

**Fig 5:**
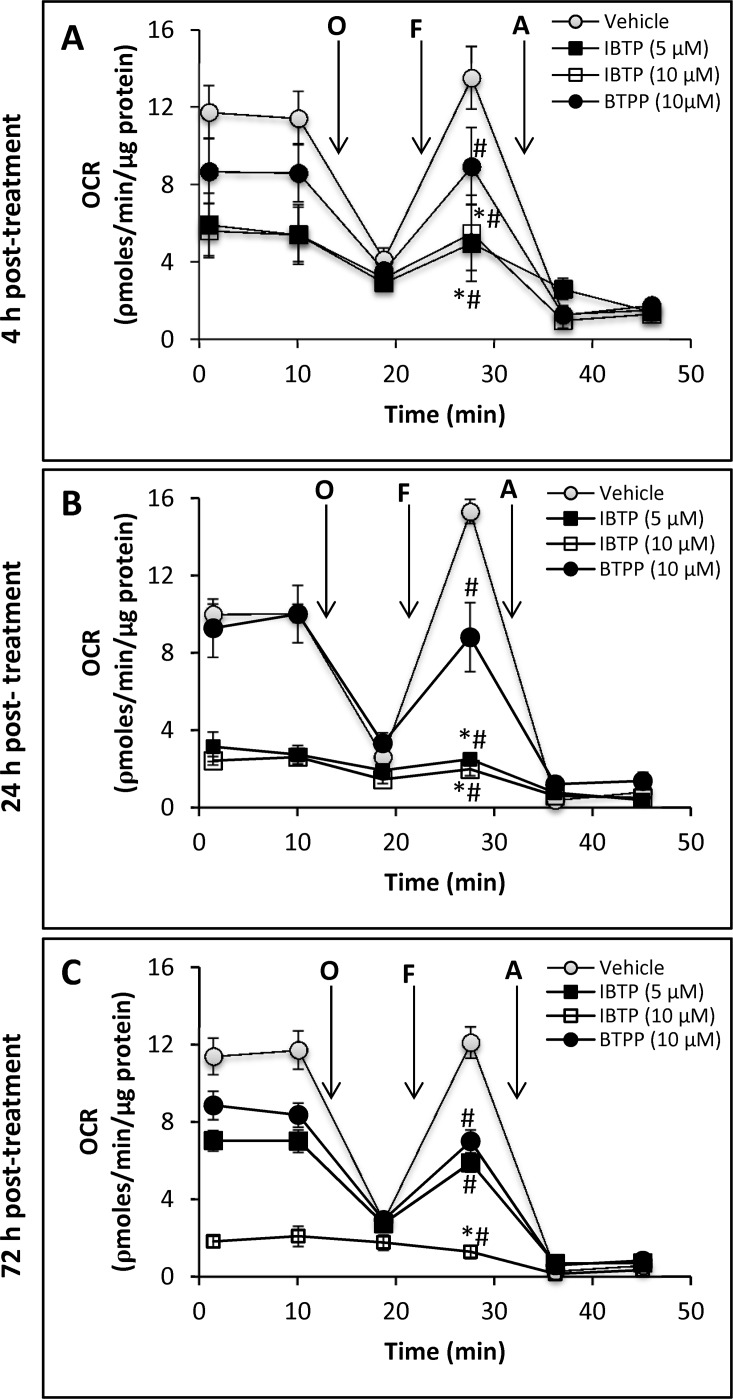
Effect of IBTP treatment on mitochondrial respiration of MB231 cells. **Panel A:** Cells plated on XF24 plates were treated with the indicated concentrations of IBTP or BTPP for 4h in 0.5% FBS-containing medium. After treatment, the medium was removed and replaced with XF assay medium (DMEM, containing 5mM glucose, 0.5% FBS, 5mM HEPES without bicarbonate) and equilibrated 1h before OCR measurement. **Panel B:** Cells plated on 6-well plates were treated with the indicated concentrations of IBTP or BTPP for 4h. After the incubation, the cells were harvested immediately by trypsinization. The harvested cells were replated in XF24 plates and allowed adhere for an additional 20h in complete medium containing 10% FBS (total 24h). The medium was removed and replaced with assay medium and equilibrated 1h before OCR measurement. **Panel C**: After 4h of IBTP or BTPP treatment, the medium was replaced with complete medium containing 10% FBS, and incubated for 48h. The cells were harvested after 48h, replated in XF24 plates and allowed adhere for an additional 20h in complete medium. The medium was replaced with assay media and incubated 1h before measurement of OCR (total duration 72h).

**Fig 6:**
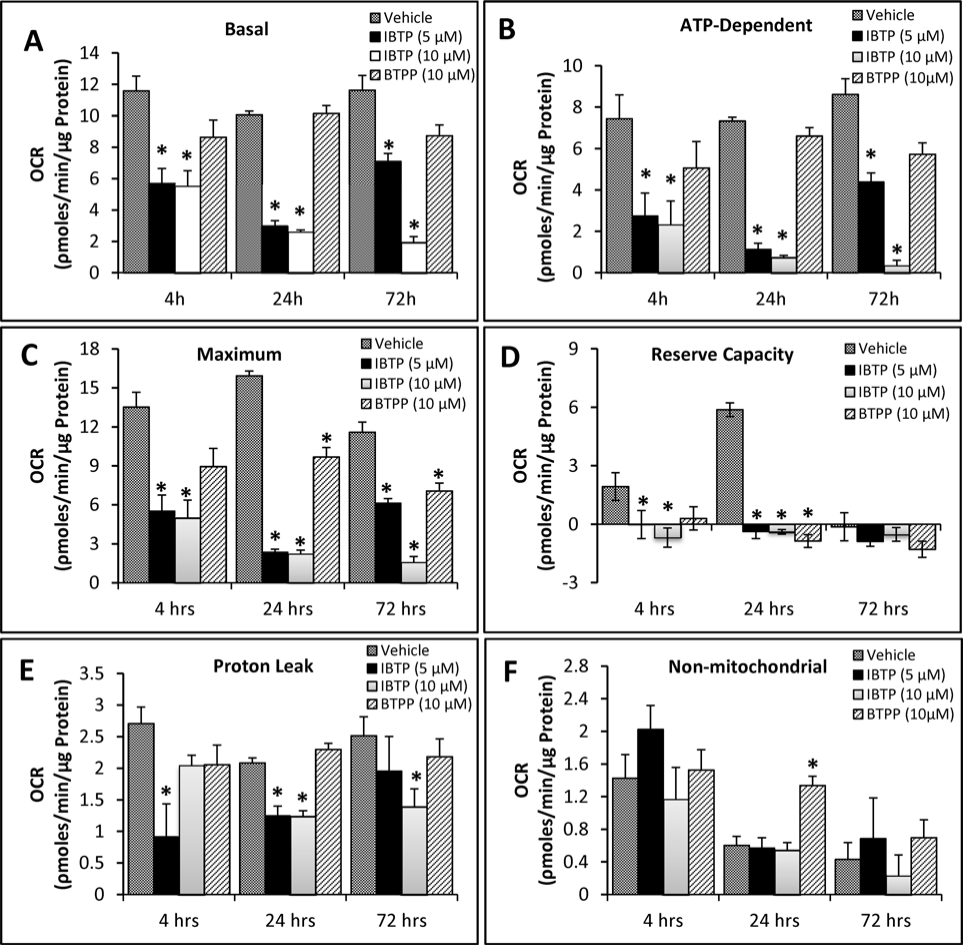
Bioenergetic parameters in MB231 cells. **Panels A-F:** Bioenergetic parameters were calculated from the OCR traces in Fig. 5, panels A-C. Values are mean ± SE obtained from 10–15 wells in two separate experiments; *p<0.05 compared to BTPP; #*p*<0.05 compared to vehicle.

As IBTP accumulates within the mitochondrion and forms protein adducts, we sought to determine the effects of IBTP on the metabolic parameters of MB231 cells over time. MB231 cells were treated with different doses of IBTP or BTPP for 4h, at which point various bioenergetic parameters were measured using the “mitochondrial stress test.” (Fig. 5A, 4h). In order to avoid differences in proliferation due to treatments for longer time points, medium was changed after treatment (4h), and cells were allowed to recover for 24h or 72h. A defined number of cells were then transferred and analyzed in the mitochondrial stress test (Fig. 5B, 24h; Fig. 5C, 72h). Treatment of MB231 cells with 5 and 10μM IBTP significantly inhibited the basal OCR at all the time points measured; however, by 72h post-treatment cells treated with 5μM IBTP began to recover. In contrast, the non-electrophilic TPP compound BTPP did not affect the basal OCR significantly at any time point studied (Fig. 6A). However, maximal OCR (24h and 72h post-treatment) and reserve capacity (24h post-treatment) were significantly decreased and non-mitochondrial OCR (24h post-treatment) was significantly increased after BTPP exposure. Thus, the triphenylphosphonium moiety has distinct effects alone in the absence of an electrophilic component, and these effects persist 24–72h post-treatment. There were no significant effects on ATP-linked OCR in cells treated with the non-reactive analog, BTPP (Fig. 6, panels A and B). After IBTP treatment, basal OCR, ATP-linked OCR, maximal OCR, and reserve capacity were all significantly decreased at 4h-, 24h-, and 72h post-treatment (Fig. 6, panels A-D). Proton leak was decreased by IBTP, and the effect recovers over time at the 5μM IBTP dose, but not at 10μM (Fig. 6, panel E). There was no significant change in non-mitochondrial OCR with IBTP (Fig. 6, panel F).

**Fig 7:**
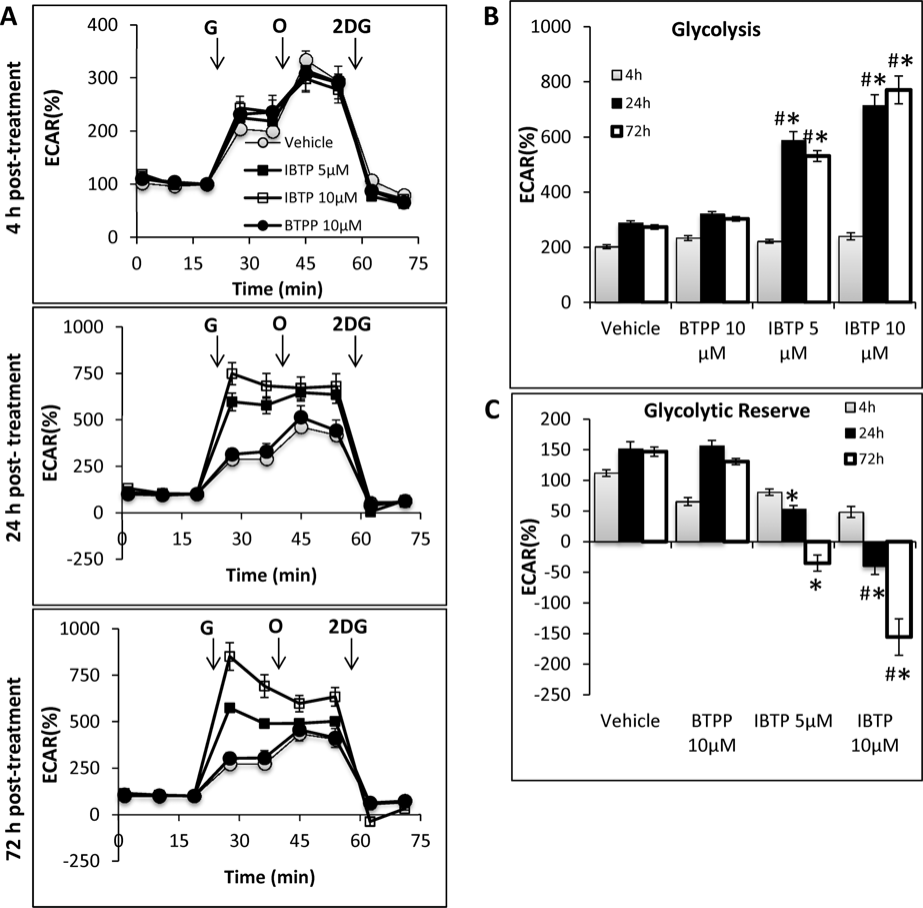
Effect of IBTP treatment on glycolysis of MB231 cells. Cells were plated, cultured, and treated as described for Fig. 5 and a glycolysis stress test was performed as described in the Materials and Methods. **Panel A:** ECAR is represented as a function of time, and the third untreated time point was used to define 100% ECAR for each group. Arrows indicate times of injection for glucose (G), oligomycin (O), and 2-deoxyglucose (2DG). **Panel B:** Rate of glycolytic flux in MB231 cells with and without BTPP or IBTP treatment. Glycolysis was defined as the %ECAR after addition of glucose (10mM). **Panel C:** Glycolytic reserve of MB231 cells with and without BTPP or IBTP treatment. Glycolytic reserve represents the difference in ECAR between glycolysis and the maximum glycolytic capacity observed after addition of oligomycin (1μM). Values are mean ± SE obtained from 10–15 wells in two separate experiments; *p<0.05 compared to BTPP; # *p*<0.05 compared to vehicle.

Due to the decreased mitochondrial metabolism observed in response to IBTP, we investigated the possibility of compensatory metabolic activity in the glycolysis pathway. Glycolytic activity was measured using extracellular flux analysis with a glycolysis stress test, as described previously [36]. The cells were cultured and treated similarly to the previous experiment for OCR, with the exception that the media was unbuffered and did not contain glucose (see Materials and Methods), allowing for the measurement of extracellular acidification rate (ECAR) in response to glucose. There were no significant differences in baseline) non-glycolytic) ECAR across groups at each time point, therefore, the baseline reading of ECAR was taken and defined as 100% for each group in subsequent analyses. Glycolysis was stimulated by the addition of glucose (Fig. 7A, “G” arrows), the glycolytic reserve capacity was determined by inhibiting mitochondrial ATP production at Complex V with oligomycin (“O” arrows), and specificity for glycolysis was determined by inhibiting flux through glycolysis with 2-deoxyglucose (“2DG” arrows). ECAR measured was specific for glycolysis because addition of 2DG returned ECAR levels to those observed in the absence of glucose. Glycolysis rate values are shown in Fig. 7B (obtained after addition of glucose), and the results indicate that no significant changes were found in any parameters at 4h, but that at 24h and 72h glycolysis was induced by IBTP (5 or 10 μM). Significant differences emerged in the glycolytic reserve capacity (Fig. 7C), which was decreased by IBTP treatment (24 and 72h post-treatment), but not BTPP. These results suggest that MB231 cells treated with IBTP for 4h, followed by 24h or 72h recovery increases glycolytic flux at the expense of the glycolytic reserve. ATP levels measured at the 24h post-treatment time point were not significantly changed by BTPP or IBTP (data not shown), indicating that the maintenance of cellular ATP can be attributed to increased glycolytic flux.

### Effect of IBTP on cell proliferation

**Fig 8:**
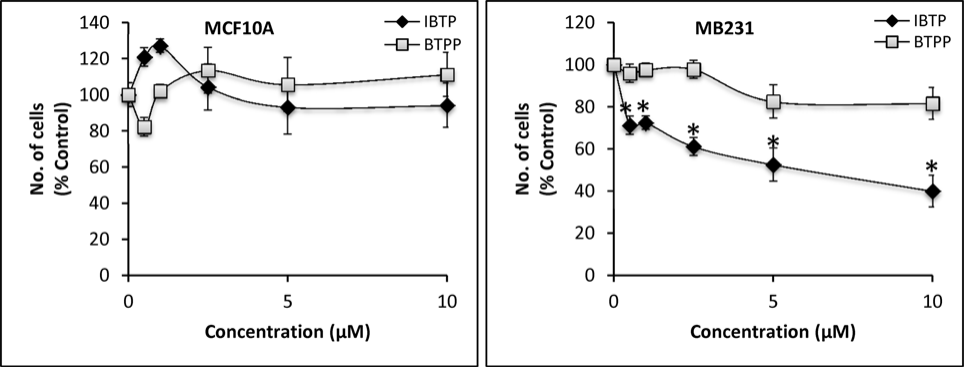
Effects of IBTP on proliferation of human breast cancer cells or non-tumorigenic breast epithelial cells. MB231 or MCF10A cells were treated with the indicated concentrations of IBTP (black diamonds) or BTPP (gray squares) for 24h. The number of cells in each group at the end of the experiment was measured. The values are mean ± SE of duplicates from two independent experiments. (*p<0.05 compared to BTPP).

Next, we sought to determine the effects of IBTP on cell proliferation of MB231 cells compared with non-tumorigenic MCF10A cells. Both cell lines were treated with concentrations ranging from 500nM to 10μM for 4h or 24h, followed by recovery for an additional 48h. The effect of IBTP on cell proliferation was analyzed by quantifying the number of cells in each well. Exposing MB231 or MCF10A cells to different doses of IBTP for short durations (4h), did not significantly affect proliferation (data not shown). However, prolonged incubation with IBTP for 24h caused significant inhibition of cancer cell proliferation (Fig. 8). IBTP inhibited the proliferation of MB231 cells dose-dependently, as demonstrated by decreased cell number, ranging from 29% (500nM) to 60% (10μM) compared to vehicle-treated cells. Under identical conditions, the nonreactive analog BTPP did not cause significant inhibition of the proliferation in MB231 cells. Significant inhibition of proliferation was also observed in another triple negative breast cancer cell line MB468 (data not shown). On the other hand, IBTP treatment did not inhibit cell proliferation significantly in MCF10A cells. Interestingly, we have previously reported that the basal OCR of MB231 cells is approximately two-fold higher than the OCR of MCF10A cells [3], suggesting that differences in mitochondrial function may render MB231 cells more susceptible to the inhibitory effects of IBTP on proliferation.

### Effect of IBTP on cell adhesion and migration

**Fig 9:**
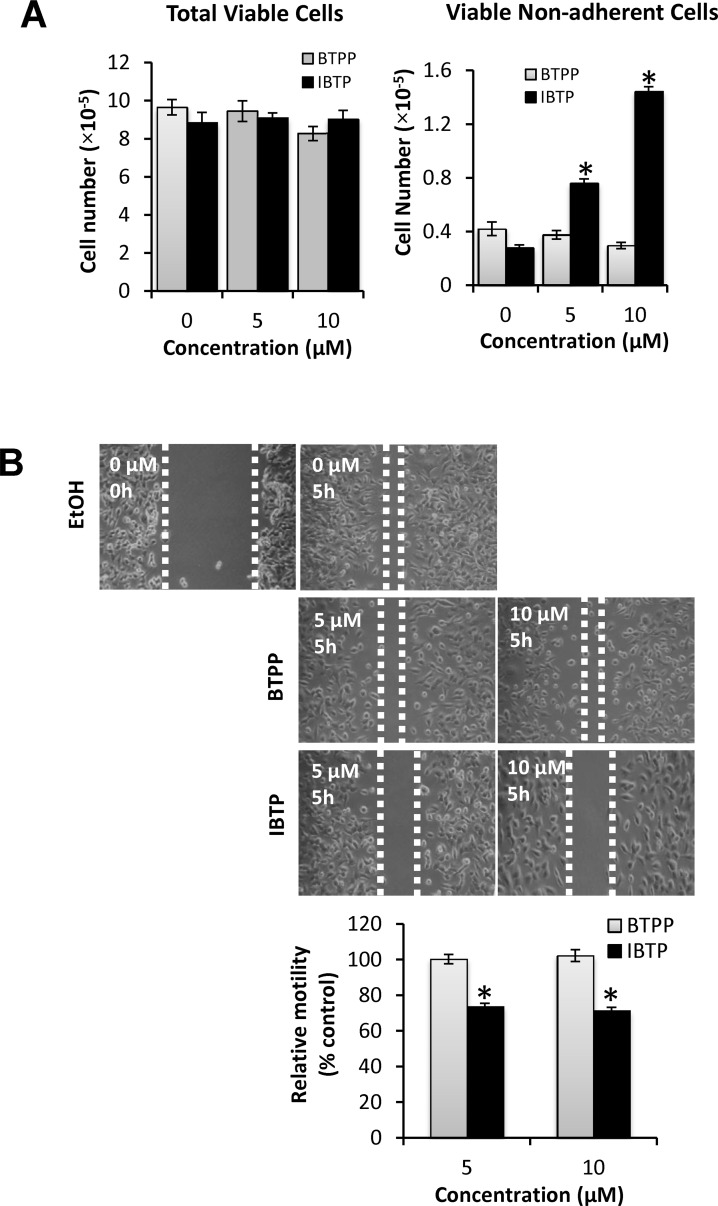
Effects of IBTP on cell attachment and migration in human breast cancer cells. MB231 cells were treated with the indicated concentrations of IBTP. At the end of the treatment, the cells were either assayed for the cells ability to attach the substratum or migration by scratch assay. **Panel A:** To determine the effect of IBTP on cell attachment, viable cells were counted (**“Total Viable Cells”**) and replated onto a 100mm tissue culture dish for 24h. At the end of the incubation, the media was collected and number of viable cells that failed to attach to the substratum was counted (**“Viable non-adherent Cells”**). The values represent the mean ± SE of triplicates from two independent experiments. (*p<0.05 compared to BTPP). **Panel B:** To determine the effect of IBTP or BTPP on migration, cells were treated with the indicated concentration of compounds for 4h. A scratch assay was performed as described in the Materials and Methods, where cells were allowed to migrate into the cell-free zone for 5h. The values represent the mean ± SE of three separate images obtained from triplicate wells. (*p<0.05 compared to BTPP).

Cancer cells are recognized for their ability to migrate away from the primary tumor and adhere at distal sites to form new colonies during metastasis. Therefore, we next examined the effect of IBTP on MB231 cell attachment and ability to migrate *in vitro*. To study the effect of IBTP on cell attachment, the cells were treated with different doses of IBTP for 4h. After treatment, cells were trypsinized, counted, and the viability was noted. All the cells from each well were transferred to new 100-mm plates and allowed to attach and grow for an additional 24h. At the end of the incubation, the media was collected and the number of viable cells in the media was determined, representing the number of viable cells that failed to attach to the substratum. Fig. 9, panel A represents total viable cells present in each group. IBTP or BTPP treatment for 4h had no effect on the viability of tumor cells (Fig. 9, Panel A: “Total Viable Cells”). However, treatment with IBTP dose-dependently inhibited the ability of MB231 cells to attach to the substratum, as indicated by increased number of viable cells floating in the medium (Fig. 9, Panel A: “Viable Non-Adherent Cells”). However, BTPP did not exhibit any significant inhibition and was comparable to vehicle treated groups.

To investigate the effect of IBTP on the migration of cancer cells, we used the scratch assay method (Fig. 9, panel B). After treatment with different doses of IBTP or BTPP, cell-free zones were created in the cell monolayer using a sterile pipet tip. After incubation for 5h, the extent to which the cells migrate into the cell-free zone was measured. Cells treated with vehicle alone migrated into the cell-free zone almost completely (Fig. 9B: EtOH), and similar results were observed with BTPP treatment (Fig. 9B: BTPP). However, IBTP treatment (either 5 or 10μM), clearly inhibited the migration of cells into the cell-free zone as seen from increased cell-free areas compared to either vehicle or BTPP treated group (Fig. 9B: IBTP).

## Discussion

Patients with breast tumors that are negative for estrogen, progesterone, and Her2/neu receptors (triple-negative) have few treatment options and are particularly susceptible to recurrence following initial therapy, resulting in high mortality rates for patients with this tumor subtype [37, 38]. Our group and others have shown that triple negative breast cancer cells exhibit higher mitochondrial metabolism than non-tumorigenic cells [3, 7], in addition to being highly glycolytic [3, 39]. The enhanced mitochondrial metabolism suggests that mitochondria-targeted compounds might provide a novel treatment strategy. In our previous study, MB231, as well as tumorigenic, metastatic MCF10A subclones (MCF10CA a.1 and MCF10A d.1α) exhibited higher energetic parameters under ambient O_2_ levels (21%). The mitochondrial energetic parameters were further increased as O_2_ approached physiological levels (4–5% O_2_), and this was shown to be hypoxia-inducible factor 1-α (HIF-1α)-dependent [3]. We have also previously shown that chemo-resistant glioma cells are more invasive and have higher mitochondrial bioenergetics than chemo-sensitive glioma cells [40]. However, it is not the case that all cancer types exhibit higher mitochondrial metabolism. There are a limited number of known tumor types which have been shown to have decreased mitochondrial metabolism due to mutations in Krebs cycle enzymes or electron transport complex subunits (e.g., pheochromocytoma, paraganglioma, familial renal cell carcinoma, glioma) [41–43]. Nevertheless, mitochondria-driven tumors have been shown *in vivo* using ^13^C-glucose metabolic analysis [44], and evidence of upregulated mitochondrial function has been shown *in situ* in human breast cancer tissue [45]. It is likely that drugs which target highly energized mitochondria may be useful in these tumor types, and will provide a novel strategy for specific cancers having augmented mitochondrial bioenergetic profiles.

Oxidation or other post-translational modifications of protein thiols can modulate the activity of enzymes and proteins, and thereby can impact important cellular functions [16]. IBTP has previously been used as a research tool to investigate the status of reduced thiols in cell culture models, isolated mitochondria, and isolated tissues from animal models of oxidative stress [27, 30]. We have previously demonstrated biological effects of IBTP in intact cultured endothelial cells [46]. IDTP has also previously been synthesized as a thiol probe, but its activity has been less well characterized [27, 30].

In the current study, we have developed and characterized a novel series of mitochondria-targeted soft electrophiles (MTSEs) and demonstrate mitochondrial protein thiol modification of these compounds in breast cancer cells (Fig. 4). MTSEs localize to mitochondria due to the presence of a lipophilic, delocalized cationic triphenylphosphonium (TPP) moiety [30]. In the present study, we report that treatment with MTSEs containing a 6-carbon or less alkyl spacer between the iodo-and phosphonium groups results in a concentration- and time-dependent accumulation of TPP-protein adducts in triple negative breast cancer cells (Figs. 2 and 3). As the lipophilicity increases with chain length, TPP compounds more readily accumulate within the mitochondria [47]. However, when the MTSE chain length was increased to 8 or 10 carbons, fewer protein adducts were observed irrespective of the dose or duration of incubation tested. At high concentrations, this may be due to destabilization of the membrane barrier or disruption of the lipid bilayer that may limit accumulation of MTSEs within the mitochondria. At lower concentrations the greater hydrophobicity and consequent greater adsorption to the matrix-facing surface of the TPP compounds, and the penetration of the longer iodoalkyl chain into the lipid bilayer may limit their access to many of the soft nucleophiles in the mitochondrial matrix. These studies demonstrate that MTSEs containing 6 carbons or less are likely to be more effective for reacting with a wider range of mitochondrial matrix protein targets, and therefore of greater utility in anti-cancer applications.

Using extracellular flux technology, it was determined that IBTP treatment resulted in profound impairment of mitochondrial function. The inhibition of mitochondrial function may be mediated through the loss of function of one or more target protein(s). Previously identified proteins shown to be modified by IBTP in rat heart mitochondria include the Krebs cycle enzymes aconitase, isocitrate dehydrogenase, and α-ketoglutarate dehydrogenase [48], which together are responsible for providing isocitrate, α-ketoglutarate, and succinyl CoA, respectively. Isocitrate dehydrogenase and α-ketoglutarate dehydrogenase also produce NADH which can then be oxidized by Complex I of the electron transport chain (ETC). Inhibition of Krebs cycle enzymes by IBTP would be expected to decrease mitochondrial oxygen consumption by limiting the production of NADH, thereby inhibiting electron flux through the ETC. Another possible explanation for the decreased mitochondrial oxygen consumption in response to IBTP is that inhibition of key metabolic proteins may cause a shift in energy production toward other metabolic pathways. In fact, our data demonstrate that IBTP treatment of cancer cells results in increased reliance on glycolysis at the expense of the glycolytic reserve capacity (Fig. 6). It is likely that dual treatment of cancer cells with IBTP and an inhibitor of glycolysis would cause energetic failure and subsequent cell death, and further studies are warranted to explore this possibility.

IBTP was also shown to have preliminary anti-cancer properties since it inhibited proliferation, attachment and migration of cancer cells. These effects were specific to the MTSE and require protein adduct formation, as BTPP did not have similar effects. Previous studies from our group have shown that the soft electrophile 15d-PGJ_2_ also inhibits cancer cell adhesion and migration due at least in part to focal adhesion disassembly and extensive F-actin reorganization at very low concentrations [49]. It is likely that the inhibitory effects of IBTP on cancer cell migration are mediated by modification of mitochondrial proteins, but the mechanism is not yet clear. A recent report demonstrates that mitochondrial superoxide production with preserved mitochondrial function increases cancer cell migration [50]. We have previously shown in other cell types that IBTP also increases reactive oxygen species [46], but in this study we show that IBTP compromises mitochondrial function and inhibits cancer cell migration, suggesting involvement of a different mechanism. Zhao *et al*. recently reported that mitochondrial function has an important role in breast cancer cell migration, and that blocking mitochondrial ATP production with oligomycin A inhibits breast cancer cell migration [51]. Our results that IBTP inhibits mitochondrial ATP production and migration in MB231 cells are consistent with this potential mechanism. Further studies are necessary to determine which protein target(s) are involved in IBTP’s effects on cancer cell migration.

In order to attribute these effects to the electrophilic nature of the compounds, we used BTPP as a mitochondria-targeted non-electrophilic control. BTPP localizes to the mitochondrion in a similar manner to IBTP [30], but does not form stable protein adducts on cysteine residues like IBTP. BTPP also does not affect the malignant phenotypes of breast cancer cells suggesting that electrophilic modification of specific mitochondrial proteins is involved in the activity of MTSEs. Interestingly, BTPP also caused some changes in mitochondrial function; however, to significantly lesser extents than MTSEs. We have previously reported this type of partial effect with TPP control molecules in studies investigating the ability of IBTP to attenuate HO-1 induction by lipid electrophiles [46]. Presumably, the transient inhibition by BTPP alone represents changes in mitochondrial function that are the result of accumulation of the triphenylphosphonium moiety within the mitochondrion, whereas the longer and more pronounced inhibition observed with IBTP treatment is the result of thiol modification. This is consistent with reports that ion fluxes alter respiration in multiple cell types. For example, mitochondrial Ca^2+^ is a positive effector of ATP synthesis [52]; however, Ca^2+^ overload results in mitochondrial dysfunction and ultimately cell death [52]. In addition, opening of the mitochondrial ATP-sensitive potassium channels and increased potassium flux has been shown to change matrix volume [53], induce Complex I-dependent ROS generation [54] [55], and inhibit mitochondrial permeability transition [56].

In summary, this study demonstrates for the first time the synthesis and characterization of MTSE analogs, as well as the demonstration that mitochondria-targeted soft electrophilic compounds decrease breast cancer cell proliferation, adhesion, migration and mitochondrial metabolism. These compounds are not overtly cytotoxic at the time points examined in this study, suggesting that they may prove to be useful for prevention of tumor recurrence and induction of cytostasis. This is an important aspect, because the main cause of death, particularly with triple negative breast cancers, is recurrence and subsequent metastasis. Therefore, development of agents such as MTSEs represents a novel treatment strategy for triple negative breast cancer where few treatment options currently exist.

## Supporting Information

S1 Fig Representative dose-dependent modification of proteins by IBTP or BTPP in MB231 cells. MB231 cells were treated with EtOH vehicle, IBTP (1–15μM), or BTPP (10 or 15μM) for 4h. At the end of treatment, cell lysates were prepared and protein adducts were visualized by Western blot analysis using an antibody directed against TPP.
(TIFF)

S2 Fig Representative crude separation of mitochondrial and cytosolic fractions. MB231 cells were treated with EtOH vehicle (C), 10μM IBTP (IB), or 10μM BTPP (BT). Mitochondrial and cytosolic fractions were obtained as described in the Materials and Methods. Relative purity was confirmed by the absence of the voltage-dependent anion channel (VDAC) in the cytosolic fractions, and its presence in the mitochondrial fractions.
(TIFF)
