# Supplementary figures and images for: A Novel Class of Mitochondria-Targeted Soft Electrophiles Modifies Mitochondrial Proteins and Inhibits Mitochondrial Metabolism in Breast Cancer Cells through Redox Mechanisms
Source: PLoS One. 2015 Mar 18;10(3):e0120460. doi: 10.1371/journal.pone.0120460 (PMC4364723; doi:10.1371/journal.pone.0120460)

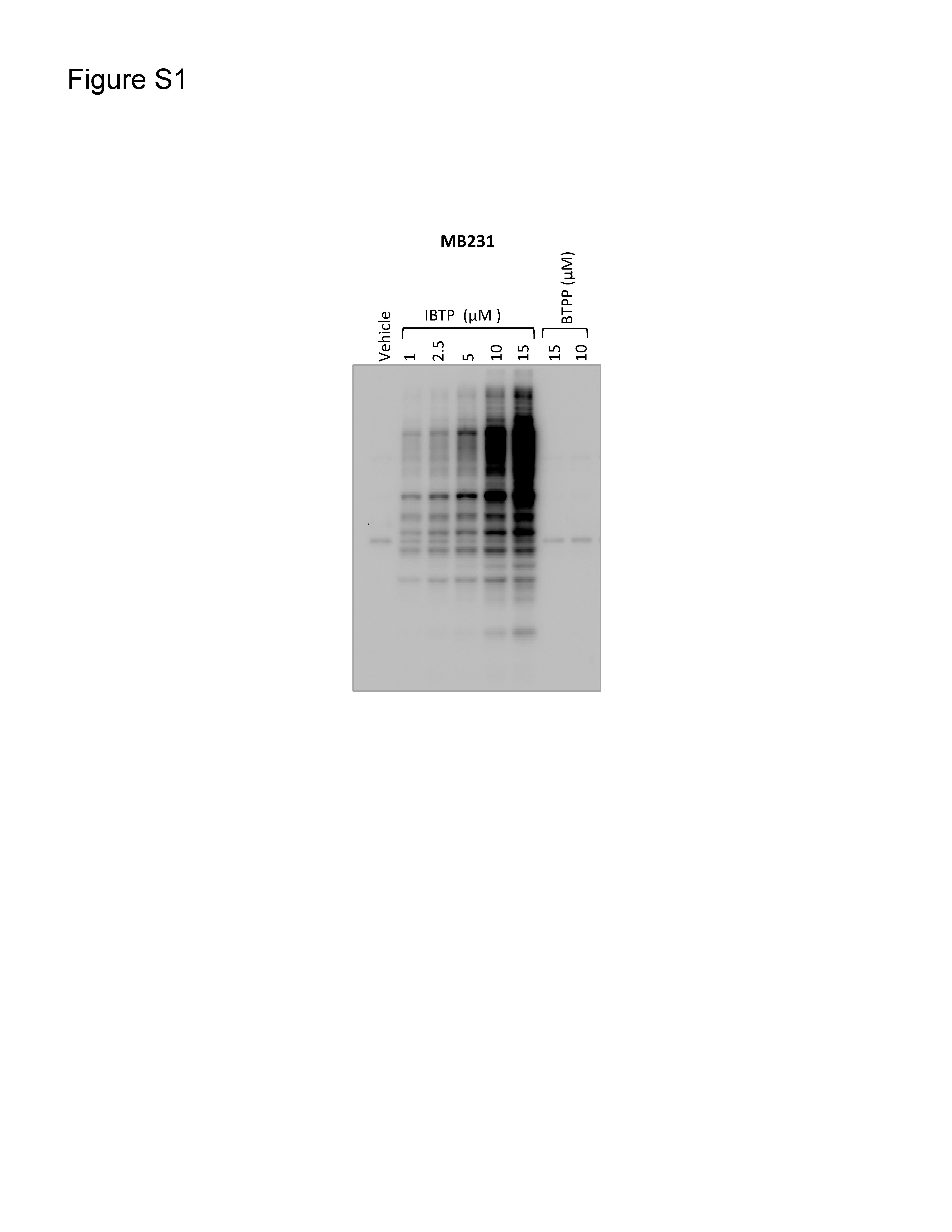

Supplement: S1 Fig — MB231 cells were treated with EtOH vehicle, IBTP (1–15μM), or BTPP (10 or 15μM) for 4h. At the end of treatment, cell lysates were prepared and protein adducts were visualized by Western blot analysis using an antibody directed against TPP. (TIFF) [file pone.0120460.s001.tiff]

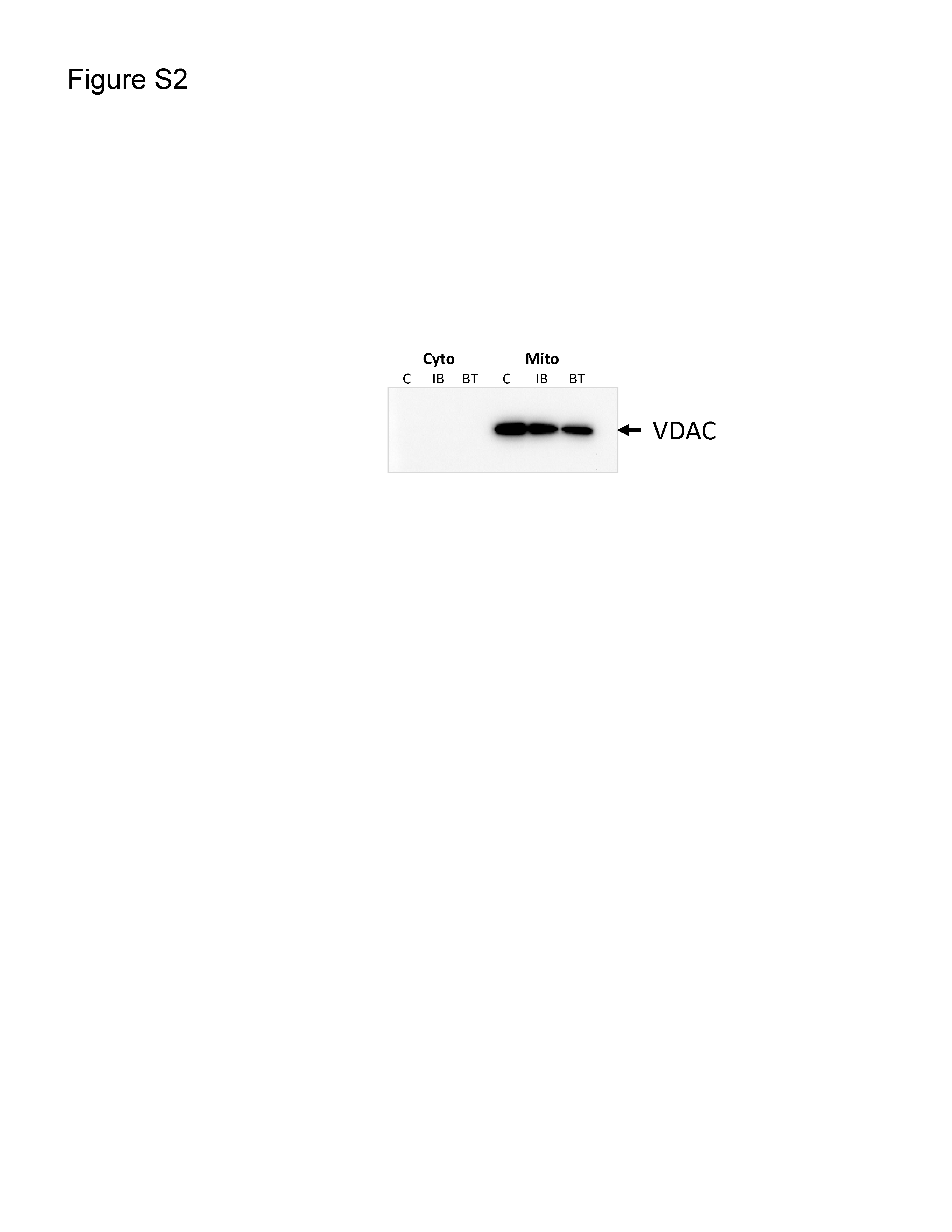

Supplement: S2 Fig — MB231 cells were treated with EtOH vehicle (C), 10μM IBTP (IB), or 10μM BTPP (BT). Mitochondrial and cytosolic fractions were obtained as described in the Materials and Methods. Relative purity was confirmed by the absence of the voltage-dependent anion channel (VDAC) in the cytosolic fractions, and its presence in the mitochondrial fractions. (TIFF) [file pone.0120460.s002.tiff]
